# Biclustering of Gene Expression Data by Correlation-Based Scatter Search

**DOI:** 10.1186/1756-0381-4-3

**Published:** 2011-01-24

**Authors:** Juan A Nepomuceno, Alicia Troncoso, Jesús S Aguilar-Ruiz

**Affiliations:** 1 Dpt. Lenguajes y Sistemas Informáticos, ETSII, University of Seville, Avd. Reina Mercedes s/n, 41012, Seville, Spain; 2 Department of Computer Science, School of Engineering, Pablo de Olavide University, Ctra. Utrera km. 1, 41013, Seville, Spain

## Abstract

**Background:**

The analysis of data generated by microarray technology is very useful to understand how the genetic information becomes functional gene products. Biclustering algorithms can determine a group of genes which are co-expressed under a set of experimental conditions. Recently, new biclustering methods based on metaheuristics have been proposed. Most of them use the *Mean Squared Residue *as merit function but interesting and relevant patterns from a biological point of view such as shifting and scaling patterns may not be detected using this measure. However, it is important to discover this type of patterns since commonly the genes can present a similar behavior although their expression levels vary in different ranges or magnitudes.

**Methods:**

Scatter Search is an evolutionary technique that is based on the evolution of a small set of solutions which are chosen according to quality and diversity criteria. This paper presents a Scatter Search with the aim of finding biclusters from gene expression data. In this algorithm the proposed fitness function is based on the linear correlation among genes to detect shifting and scaling patterns from genes and an improvement method is included in order to select just positively correlated genes.

**Results:**

The proposed algorithm has been tested with three real data sets such as Yeast Cell Cycle dataset, human B-cells lymphoma dataset and Yeast Stress dataset, finding a remarkable number of biclusters with shifting and scaling patterns. In addition, the performance of the proposed method and fitness function are compared to that of CC, OPSM, ISA, BiMax, xMotifs and Samba using Gene the Ontology Database.

## 1 Background

DNA microarray technology measures the gene expression level of thousand of genes under multiple experimental conditions [1]. After several preprocessing steps well-known as *low level microarray analysis *a microarray can be represented as a numerical matrix where rows correspond to different genes and columns to experimental conditions. The row vector of a gene is called the *expression pattern *of the gene and a column vector is called the *expression profile *of the condition. *High level microarray analysis *uses data mining techniques in order to analyze the huge volume of all this biological information [2]. In this field, an important problem is to discover transcription factors which determine that a group of genes are co-expressed. Thus, the goal of *Biclustering *techniques is to discover groups of genes with the same behavior under a specific group of conditions.

Biclustering was considered in the seventies and it was proven to be a NP-hard problem [3]. It can be also found in the literature with other names such as Subspace Clustering [4] or Co-clustering [5]. Several algorithms have been proposed and recently published reviews can be found in [6-8]. In the context of microarray analysis, biclustering was firstly considered by Cheng and Church in 2000. Cheng and Church (CC) algorithm [9] is a greedy iterative search method and consists in building a bicluster adding or removing rows or columns iteratively, thus, improving its quality which is measured with the *Mean Squared Residue *(MSR). The MSR is based on the sum of the squared residues which measure how adequate each expression value is, in comparison with the rest of values of the bicluster (see [9] for more details). The FLOC algorithm [10] improved the method presented in [9] by obtaining a set of biclusters simultaneously and by incorporating the processing of missing values. In [11] an iterative hierarchical clustering is separately applied to each dimension and biclusters are built by means of the combination of the obtained results for each dimension. In the ISA algorithm [12] a simple linear model for gene expression is used assuming a normally distributed expression level for each gene or condition in a specific way. SAMBA [13] executes an exhaustive biclusters enumeration by means of a bipartite graph-based model and later a greedy approach adds or removes nodes in order to find maximum weight subgraphs. In [14] an exhaustive biclusters enumeration algorithm is proposed with the OPSM algorithm. Spectral biclustering [15] uses techniques from linear algebra, concretely eigenvectors calculus, to identify bicluster structures from the input data. The Plaid Model [16] is a statistical modelling approach which represents the input matrix as a superposition of layers where each layer corresponds to a bicluster. The BiMax algorithm [17] discretizes the data set by using binary values and it is recursively applied until a submatrix with only one value is detected. Geometrical characterization of biclusters are used for discovering patterns [18,19]. These techniques use image processing in order to search for hyperplanes which represent biclusters. There is a group of biclustering algorithms based on metaheuristics such as evolutionary approaches [20,21], multiobjective evolutionary approaches [22,23], Simulated Annealing [24], Particle Swarm Optimization [25], greedy randomized adaptive search [26], Estimation of Distribution Algorithms [27] or Memetics Algorithms [28]. All these algorithms used the MSR as a part of their fitness function. Although the MSR is commonly used as quality criterion, some interesting patterns from a biological point of view might not be detected with such measure. The MSR is effective for recognizing biclusters with shifting patterns but not some patterns with scaling trends, in spite of representing quality patterns. A group of genes has a *shifting pattern *when the expression values vary in the addition of a fixed value for all the genes. A group of genes has a *scaling pattern *when the expression values vary in the multiplication of a fixed value for all the genes. Aguilar-Ruiz [29] proved that the MSR is not a good measure in order to discover patterns in data when the variance of gene values is high, that is, when the genes present scaling patterns.

Other algorithms are designed to work with time series gene expression data. In this kind of data, the biclusters can be restricted to those with contiguous columns. This constraint becomes the biclustering problem in a tractable problem. CCC-Biclustering [30] finds coherent biclusters with maximal contiguous columns in linear time. First, the algorithm discretizes the matrix and then it works with string processing techniques based on suffix trees. This algorithm is not robust as regards errors in gene values due to the discretization process, the microarray experiment, etc. e-CCC-Biclustering [31] is a robust extension of the CCC-Biclustering where approximate expression patterns such as scaling patterns can be found and several measures to compute the committed errors in these patterns are proposed.

The gene expression level under a set of conditions can be seen as values of a discrete random variable. Thus, the linear dependency between two genes can be studied by using the correlation coefficient between two random variables. In this paper, this fact has motivated the use of the proposed measure based on correlations among genes [32,33]. Several correlation-based measures have been proposed in [4,34,35]. In [4] biclusters are characterized as hyperplanes in a high dimensional space using the definition of correlation and, therefore, the problem is transformed into the search for groups of points embedded in hyperplanes. In [34] the correlation coefficient is used for forming biclusters with a greedy algorithm. In [35] an enumeration algorithm based on a tree structure for biclustering is presented and it uses an evaluation function based on the Spearman's rank correlation.

A Scatter Search algorithm for biclustering is presented in this paper. Scatter Search is a population-based method that emphasizes systematic processes against random procedures. Thus, the generation of the initial population is not random but a generation method based on diversification [36] is used to generate a set of diverse initial solutions. Moreover, Scatter Search includes an improvement method with the aim of exploiting the diversity provided by the generation and combination method. The linear correlation among genes is included in the fitness function to evaluate the quality of biclusters in the Scatter Search, which improves the localization of shifting and scaling patterns.

## 2 Description of the algorithm

Scatter Search [36] is a population-based optimization metaheuristic which has recently been applied to combinatorial and nonlinear optimization problems. Optimization algorithms based on populations are search procedures where a set of individuals that represent trial solutions evolve in order to find optimal solutions of the problem. Scatter Search uses strategies to diversify and intensify the search in order to avoid local minima and to find quality solutions and, on the opposite to other evolutionary heuristics, it emphasizes systematic processes against random procedures.

Basically, the optimization process consists in the evolution of a set called *Reference Set*. This set is initially built with the best solutions from the population, according to the value of their fitness function, and the most scattered ones from the population regarding the previous best solutions. This set is updated by using the *Combination Method *and the *Improvement Method *until it does not change. When the *Reference Set *is stable, that is, after applying the combination and improvement methods it contains the same solutions that the reference set at the previous iteration, then it is rebuilt again. That is, the building of the *Reference Set *is based on quality and diversity, but its updating is only guided by quality. Thus, diversity is introduced in the evolutionary process when the initial population is generated and, mainly, when the reference set is rebuilt at each step. The search intensification is due to the improvement method where the solutions are improved by exploiting the knowledge of the problem.

The pseudocode of the proposed Scatter Search for biclustering is presented in Algorithm 1. The Scatter Search process is repeated *numBi *times where *numBi *is the number of biclusters to be found and the best solution of the reference set is stored in a set called *Results *for each iteration. Thus, the *Results *set is formed by *numBi *biclusters and it is the output of the Algorithm 1. The Scatter Search mainly consists in a diversification generation method to generate the initial population, a combination method to create new offspring and an improvement method to intensify the search. All theses steps of the Scatter Search are detailed as follows.

**Algorithm 1 **Scatter Search Algorithm for Biclustering

**INPUT **microarray *M *, number of biclusters to be found *numBi*, maximum number of iterations *numIter*, size of the initial population and size *S *of the reference set.

**OUTPUT **Set *Results *with *numBi *biclusters.

begin

   *num *← 0, *Results *← ϕ

   **while **(*num *<*numBi*) **do**

      Initialize population *P*

      *P *← Improvement Method **(***P ***)**

      **//**Building Reference Set

      *R*_1 _← *S*/2 best biclusters from *P *(according to the fitness function)

      *R*_2 _← *S*/2 most scattered biclusters, regarding *R*_1 _, from *P *\ *R*_1 _(according to a distance).

      *RefSet *← (*R***_1 _**∪ *R*_2 _)

      *P *← *P *\ *RefSet*

      //Initialization

      stable ← FALSE, *i *← 0

      **while **(*i *<*numIter*) **do**

         **while **(NOT stable) **do**

            *A *← *RefSet*

            *B *← Combination Method(*Ref Set*)

            *B *← Improvement Method(*B*)

            *RefSet *← *S *best biclusters from *Ref Set *∪ *B*

            **if **(*A *= *RefSet*) then

               *stable *← *TRUE*

            **end if**

         **end while**

         //Rebuilding Reference Set

         *R*_1 _← *S*/2 best biclusters from *RefSet*

         *R*_2 _← *S*/2 most scattered biclusters from *P *\ *R*_1_

         *RefSet *← (*R*_1 _∪ *R*_2 _)

         *P *← *P *\ *RefSet*

         *i *← *i *+ 1

      **end while**

      //Storage in Results

      *Results *← the best one from *RefSet*

      *num *← *num *+ 1

   **end while**

end

### 2.1 Initialization phase

Formally, a microarray is a real matrix *M *composed of *N *genes and *L *conditions. The element (*i, j*) of the matrix means the level of expression of gene *i *under the condition *j*. A bicluster *B *is a submatrix of the matrix *M *composed of *n ≤ N *rows or genes and *l ≤ L *columns or conditions. Biclusters are encoded by binary strings of length *N *+ *L*. Each of the first *N *bits of the binary string is related to the genes and the remaining *L *bits to the conditions. For example, the string 0010110000|01100 represents a bicluster of a microarray with ten genes, {*g_i_*}_1≤*i*≤10_, and five conditions, {*c_j _*}_1≤*j*≤5 _. This string encodes the bicluster composed by the genes *g*_3_, *g*_5 _and *g*_6 _and the conditions *c*_2 _and *c*_3_.

The initial population is generated with solutions as diverse as possible. Thus, the diversification generation method [36] takes a binary string, *x_i _*with *i *= 1, . . . , *n *where *n *is the number of bits, as a seed solution and generates solutions ${x^{\prime }}_{i}$ by following the rule:

(1) $${x^{\prime }}_{1+kh}=1-x_{1+kh}\;\mathrm{for}\;k=0,1,2,3,...,\text{⌊}n/h\text{⌋}$$

where ⌊*n/h*⌋ is the largest integer less or equal than *n/h *and *h *is an integer less than *n/*5. All the remaining bits of x′ are equal to that of *x*.

After generating all the posible solutions with that seed, if more solutions are needed, the diversification generation method is applied again by using the last solution as new seed.

### 2.2 Biclusters Evaluation: Fitness Function

The most difficult biclusters to find are those that present jointly shifting and scaling patterns. The aim of this work is to discover this type of biclusters. Two genes show a shifting and scaling pattern if they are described from (2).

(2) $${\text{g}}_{\text{Y}}\;=\;\alpha {\text{g}}_{\text{X}}+\beta \;\alpha ,\beta \in \mathbb{R}$$

Consequently, two genes with shifting and scaling patterns are linearly dependent and therefore a measure based on correlations can be a good fitness function to find biclusters with these patterns.

The correlation coefficient between two variables *X *and *Y *measures the grade of linear dependency between them. It is defined by:

(3) $$\rho (X,Y)=\frac{cov(X,Y)}{\sigma_{X}\sigma_{Y}}=\frac{\sum_{i}^{n}\left(x_{i}-\bar{x}\right)\left(y_{i}-\bar{y}\right)}{n\sigma_{X}\sigma_{Y}}$$

where *cov*(*X, Y *) is the covariance of the variables *X *and *Y *, $\bar{x}$and $\bar{y}$ are the mean of the values of the variables *X *and *Y *and *σ_X _*and *σ_Y _*are the standard deviations of *X *and *Y *, respectively.

Given a bicluster *B *composed by *N *genes, *B *= [*g_1_, . . . , g_N _*], the average correlation of *B*, *ρ*(*B*), is defined as follows,

(4) $$\rho (B)=\frac{1}{\left(\begin{array}{c} N\\ 2 \end{array}\right)}\sum_{i=1}^{N-1}\sum_{j=i+1}^{N}\rho (g_{i},g_{j})$$

where *ρ*(*g_i_, g_j _*) is the correlation coefficient between the gene *i *and the gene *j*. Due to *ρ*(*g_i_, g_j _*) = *ρ*(*g_j_, g_i_*), therefore, only $\left(\begin{array}{c} N\\ 2 \end{array}\right)$ elements have been considered.

**Figure 1:**
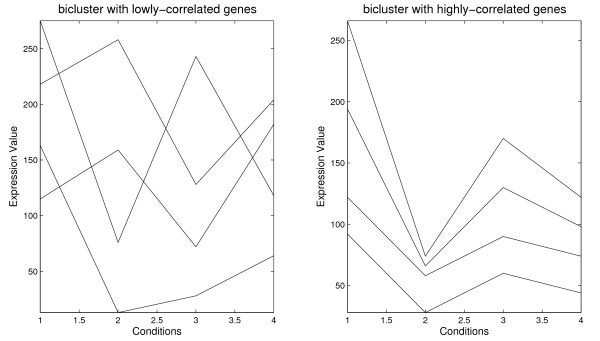
**Correlation among genes**. Biclusters with lowly-correlated genes and highly-correlated genes $$\begin{array}{cc} \left[\begin{array}{cccc} 115 & 159 & 72 & 182\\ 163 & 13 & 28 & 64\\ 218 & 258 & 128 & 204\\ 275 & 76 & 243 & 118 \end{array}\right]\Rightarrow \rho (B)=0.003 & \left[\begin{array}{cccc} 194 & 66 & 130 & 98\\ 92 & 28 & 60 & 44\\ 266 & 74 & 170 & 122\\ 122 & 58 & 90 & 74 \end{array}\right]\Rightarrow \rho (B)=1 \end{array}$$

Figure 1 presents a bicluster with lowly-correlated genes and a bicluster with highly-correlated genes. It can be observed that the bicluster with perfect shifting and scaling patterns has an average correlation of 1 while that the bicluster without patterns has an average correlation close to 0 (concretely 0.003).

In this work, biclusters with highly-correlated genes and high volume are preferred. Therefore, the fitness function used to evaluate the quality of biclusters is defined by:

(5) $$f(B)=(1-\rho (B))+\sigma_{\rho }+M_{1}\left(\frac{1}{nG}\right)+M_{2}\left(\frac{1}{nC}\right)$$

where *nG *and *nC *are the number of genes and conditions of the bicluster *B*, respectively, *M*_1 _and *M*_2 _are penalty factors to control the volume of the bicluster *B*, and *σ_ρ _*is the standard deviation of the values *ρ*(*g_i_, g_j _*) from (4). The standard deviation is included in order to avoid that the value of the average correlation can be high for a bicluster and this bicluster can contain several non-correlated genes with the remaining ones of the bicluster. Best biclusters are those with the lowest value for the fitness function. Thus, it has been considered (1 *− ρ*(*B*)) to identify biclusters with highly-correlated genes.

Moreover, this measure is robust to noise since genes showing noise but with shifting patterns can present a high correlation although scaling patterns are not involved.

### 2.3 Improvement Method

Scatter Search uses improvement methods when the solutions have to fulfill some constraints or simply to improve them in order to intensify the search process. This method depends on the problem under study and usually it consists in classical local searches for continuous optimization problems.

The goal of this work is to find biclusters with shifting and scaling patterns. Thus, biclusters with positively-correlated genes are only searched for. Therefore, the proposed improvement method aims at extracting positively-correlated genes either from biclusters of the initial population or from biclusters obtained by the combination method. The pseudocode of the improvement method is presented in the Algorithm 2.

**Figure 2:**
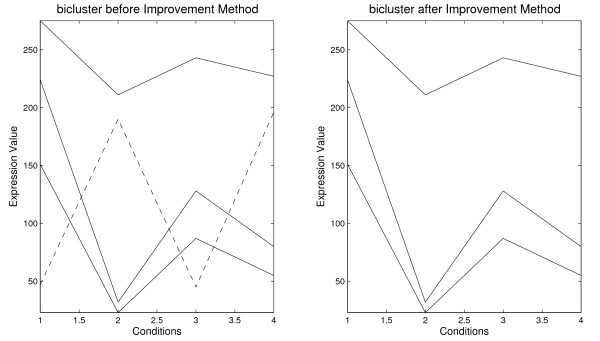
**Improvement Method**. Bicluster before and after applying the Improvement Method $$\begin{array}{cc} \left[\begin{array}{cccc} 151 & 23 & 87 & 55\\ 47 & 190 & 45 & 196\\ 224 & 32 & 128 & 80\\ 275 & 211 & 243 & 227 \end{array}\right]\Rightarrow \rho (B)=0.083 & \left[\begin{array}{cccc} 151 & 23 & 87 & 55\\ 224 & 32 & 128 & 80\\ 275 & 211 & 243 & 227 \end{array}\right]\Rightarrow \rho (B)=1 \end{array}$$

Figure 2 presents a bicluster composed by four genes: three highly-correlated genes and a gene negatively correlated with the remaining. The average correlation for this bicluster is equal to 0.0083 and after applying the improvement it is equal to 1. Thus, the volume of biclusters is decreased by removing the negatively-correlated genes but the average correlation of the new bicluster will be greater than that of the original bicluster when the improvement method is applied.

### 2.4 Building of the reference set

The reference set is initially built with the best solutions, according to the value of their fitness function, and the most scattered ones from the initial population regarding the previous best solutions. The

**Algorithm 2 **IMPROVEMENT METHOD

**INPUT **Bicluster *B *= [*g*_1_*, . . . , g_N _*]

**OUTPUT **Bicluster *B′ *⊆ *B *such that *ρ*(*g_i_, g_j _*) *≥ *0 ∀*g_i_, g_j _*∈ *B′*

begin

   *i ← *1, *B′ ← *{*g_i _*}, *R ← *{}

   **while **(i <*N *) **do**

      *j ← i *+ 1

      **while **(*j ≤ N *) **do**

         **if **(*ρ*(*g_i_, g_j _*) > 0) **then**

            **if **(*g_j _*∉ *R*) **then**

               *B′ ← B′ *∪ {*g_j _*}

            **end if**

         **else**

            *R ← R *∪ {*g_j _*}

         **end if**

         *j ← j *+ 1

      **end while**

      *i ← i *+ 1

   **end while**

end

*Hamming *distance is used to measure the distance among biclusters in this work. After getting the stability of the reference set in the updating process, it is rebuilt to introduce diversity in the search process. Thus, the reference set is rebuilt with the best biclusters from the updated reference set, according to the fitness function, and the most distant solutions from the initial population regarding the previously chosen best solutions.

The initial population has to be updated too in the evolutionary process by removing solutions which have already been considered in the building or rebuilding of the reference set. When the initial population is empty, a new population is created by using the diversification generation method previously explained in Section 2.1.

### 2.5 Combination method and reference set updating

New solutions are introduced in the search process by the combination method. Two solutions are combined by using an uniform crossover operator and a new one is generated. All pairs of biclusters in the reference set are combined, generating thus, S * (*S *- 1)*/*2 new biclusters where *S *is the size of the reference set. This crossover operator generates randomly a mask and the child is composed of values from the first parent when there is a 1 in the mask, and from the second parent when there is a 0.

After combining all pairs of biclusters, the best solutions from the joining of the previous reference set and the new solutions are chosen. Hence, best solutions according to the value of their fitness function remain in the reference set.

## 3 Experiments

The proposed algorithm has been applied to three real data sets in order to study its performance. The first data set (Yeast) is the *yeast Saccharomyces cerevisiae *cell cycle expression, with 2884 genes and 17 experimental conditions presented by Cho [37]. The second one (Lymphoma) is the *human B-cells lymphoma *expression data with 4026 genes and 96 conditions [38]. These two data sets are available in [9] where original data were processed. The third data set (GaschYeast) is the *yeast Saccharomyces cerevisiae *Stress conditions expression provided by Gasch [39] with 2993 genes and 173 conditions. This data set was used in [17] where can be downloaded as supplementary data.

The inner parameters of the Algorithm 1 are as follows: 20 for the maximum number of iterations of the Scatter Search, 10 for the size of the reference set, 200 for the number of solutions of the initial population and 100 for the number of biclusters to be found for each run. *M*_1 _and *M*_2 _parameters are weights in the fitness function in order to drive the search depending on the required size of biclusters. High values of *M*_1 _and *M*_2 _may be used when biclusters with a lot of genes and conditions are desired. Results for Yeast and Lymphoma data set have been obtained with values *M*_1 _= 1 and *M*_2 _= 1. Results for GaschYeast data set have been presented for *M*_1 _= 1 and *M*_2 _= 1 and for *M*_1 _= 10 and *M*_2 _= 10 to show the influence of these parameters on the volume of the biclusters.

### 3.1 Results

**Figure 3:**
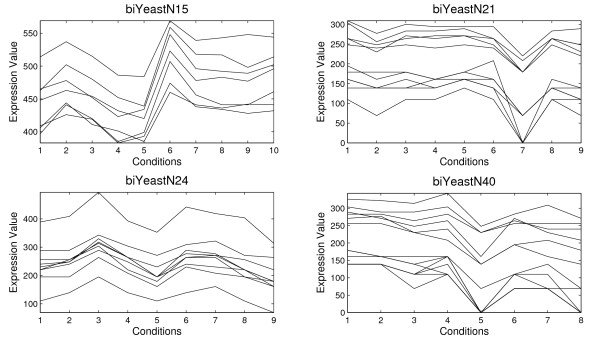
**Results for Yeast data set**. Several biclusters found by the Algorithm 1 from Yeast data set

**Figure 4:**
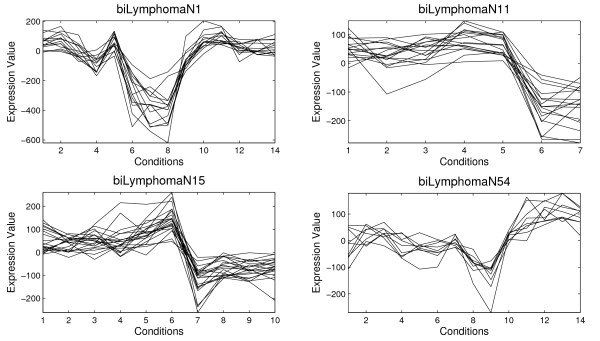
**Results for Lymphoma data set**. Several biclusters found by the Algorithm 1 from Lymphoma data set

**Figure 5:**
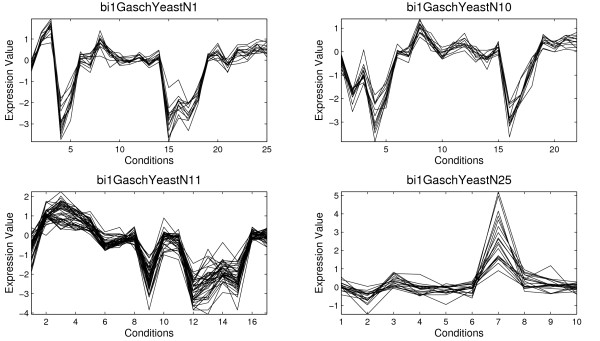
**Results for Gasch Yeast data set (*M*_1 _= 1, *M*_2 _= 1)**. Several biclusters found by the Algorithm 1 from Gasch Yeast data set obtained for values *M*_1 _= 1 and *M*_2 _= 1

**Figure 6:**
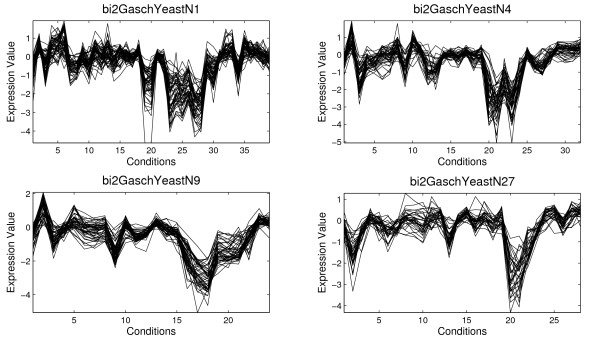
**Results for Gasch Yeast data set (*M*_1 _= 10, *M*_2 _= 10)**. Several biclusters found by the Algorithm 1 from Gasch Yeast data set obtained for values *M*_1 _= 10 and *M*_2 _= 10

Table 1 shows the information for four biclusters selected among 100 biclusters obtained by the application of the proposed Scatter Search and the average of the 100 biclusters (in bold). For each bicluster an identifier of the bicluster, the number of genes, the number of conditions, the volume, the average correlation, *ρ*(*B*), and the standard deviation, *σ*(*B*), are presented. The MSR and the variance of gene values are reported too in order to establish a comparison of the quality of biclusters with other algorithms. The variance of gene values measures how different the values of the gene expression level are. Figure 3 and 4 present the four biclusters for Yeast and Lymphoma data set, respectively, which are reported in Table 1. Figure 5 and 6 depict biclusters from GaschYeast data set. The biclusters *bi1-GaschYeastN1*, *bi1-GaschYeastN10*, *bi1-GaschYeastN11 *and *bi1-GaschYeastN25 *in Figure 5 have been obtained for values *M*_1 _= 1 and *M*_2 _= 1 and the biclusters *bi2-GaschYeastN1*, *bi2-GaschYeastN4*, *bi2-GaschYeastN9 *and *bi2-GaschYeastN27 *in Figure 6 for values *M*_1 _= 10 and *M*_2 _= 10. It can be noted that the greater the penalty values are, the greater the volume of the obtained biclusters is. The motivation for taking the values of the parameters *M*_1 _= *M*_2 _= 1 is to find biclusters with a low number of genes in order to show visually the shifting and scaling patterns. However, the main goal is to find groups of genes sharing the same GO terms, therefore, it is more adequate to search for biclusters with a high number of genes. Thus, parameters *M*_1 _= *M*_2 _= 10 have been considered to achieve biclusters with a higher volume.

**Table 1:** Information about biclusters found by the Algorithm 1

**Id bi**.	Genes	Conditions	Volume	*ρ*(*B*)	*σ*(*B*)	MSR	Genes Variance
biYeastN15	7	10	70	0.95	0.56	59.2	882.8
biYeastN21	11	9	99	0.92	0.47	205.2	1190.5
biYeastN24	9	9	81	0.92	0.45	142.9	1344.8
biYeastN40	13	8	104	0.89	0.45	368.2	2185.4
**biYeast**	22.27	6.46	133.1	0.90	0.48	321.0	1508.7

biLymphomaN1	14	14	196	0.92	0.43	3719.2	29180.0
biLymphomaN11	17	7	119	0.92	0.50	1607.9	10317.6
biLymphomaN15	21	10	210	0.86	0.43	1818.4	8351.2
biLymphomaN54	9	14	126	0.82	0.45	1292.6	6108.0
**biLymphoma**	10.81	11.53	123.7	0.85	0.45	2593.3	11643.07

bi1-GaschYeastN1	13	25	325	0.96	0.42	0.08	1.51
bi1-GaschYeastN10	12	22	264	0.95	0.48	0.06	1.19
bi1-GaschYeastN11	41	17	697	0.93	0.34	0.15	1.67
bi1-GaschYeastN25	19	10	190	0.93	0.43	0.19	0.89
**bi1-GaschYeast**	16.36	14.08	237.6	0.89	0.43	0.32	1.50

bi2-GaschYeastN1	54	39	2106	0.82	0.32	0.22	1.00
bi2-GaschYeastN4	43	32	1376	0.84	0.45	0.18	1.02
bi2-GaschYeastN9	48	24	1152	0.87	0.41	0.17	1.18
bi2-GaschYeastN27	33	28	924	0.84	0.39	0.13	0.72
**bi2-GaschYeast**	46.69	27.69	1269.4	0.72	0.34	0.38	1.02

Results from Yeast and Lymphoma data set for values *M*_1 _= 1 and *M*_2 _= 1 (four biclusters and the average of the 100 biclusters obtained). Results from GaschYeast for values *M*_1 _= 1 and *M*_2 _= 1, and for *M*_1 _= 10 and *M*_2 _= 10.

### 3.2 Discussion

Four biclusters represented in Figure 3 have a high value for the average correlation (see Table 1). It can be observed that shifting and scaling patterns can clearly be appreciated in all of them. Most of papers which use the MSR as the fitness function consider that a bicluster is good for Yeast data set if its MSR is less than 300 [9,21]. This value is data set dependent because it depends on the mean and the standard deviation of the values of the expression matrix. From this point of view, *biYeastN15*, *biYeastN21 *and *biYeastN24 *are good biclusters but *biYeastN40 *is not a good bicluster due to its high value for the average correlation. Note that the value for the gene variance in *biYeastN40 *is high compared to that of other biclusters.

Four biclusters represented in Figure 4 for Lymphoma data set show a group of genes with similar behavior and high values for the average correlation (see Table 1). However, these biclusters are not considered good for some authors because the MSR are higher than 1200 [9,21]. This value is data set dependent too as it was previously commented for Yeast data set. Note that the bicluster with lowest value for MSR is *biLymphomaN54 *(1292.6), and however this bicluster has the lowest value for the average correlation (0.82). It was proved in [29] that MSR is not precise enough in order to discover shifting and scaling patterns. Biclusters with these patterns with high values for the gene variance are not detected by the algorithms that use the MSR as fitness function. Results reported for theses cases make evident this situation.

Figure 5 and 6 show biclusters for GaschYeast data set. It can be observed the effect of the penalty parameters on the number of genes and conditions. The higher values for *M*_1 _and *M*_2_, the higher volume is. From a geometrical point of view all the results present genes with similar behavior. For example, scaling patterns can be very clearly observed in *bi1-GaschYeastN25 *since the shape of genes between conditions 6 and 8 is the same although all genes increase their expression levels with different intensity. Moreover, the value for the average correlation shows biclusters from GaschYeast with highly-correlated genes. The values for MSR and gene variance vary in a different range of values to the other two data set due to the previous preprocessing.

It should be noted that all biclusters present shifting and scaling patterns, and therefore, a high value for the average correlation. Moreover, the standard deviation is low, that is, the correlation coefficients of each pair of genes have similar values and close values to the average correlation of the bicluster. Therefore, all biclusters with a high average correlation found by the proposed Scatter Search do not contain non-correlated genes.

### 3.3 Comparative analysis

The performance of the Algorithm 1 has been compared with biclustering methods such as CC [9], OPSM [14], ISA [40], BiMax [17], xMotifs [41] and Samba [13] for the GaschYeast data set and CCC-Biclustering [30] for Yeast data set. Also, random biclusters have been generated as naive reference method. Following the methodology in [17] the performance of all algorithms is evaluated biologically with the percentage of biclusters enriched by any Gene Ontology Consortium (GO) category at different levels of significance. GO [39] is used to investigate if a group of genes belonging to a bicluster presents significant *enrichment *with respect to a specific GO term. There are different tools to analyze GO term enrichment. The AGO [42] tool recently published has been used to study the percentage of significant biclusters obtained by the different algorithms. The enrichment of each group of genes with respect to a specific GO term is established by the p-value. A bicluster is said to be *overrepresented *in a functional category if its p-value is small. The comparison criterion among several algorithms is the percentage of overrepresented biclusters in one or more GO annotation.

**Figure 7:**
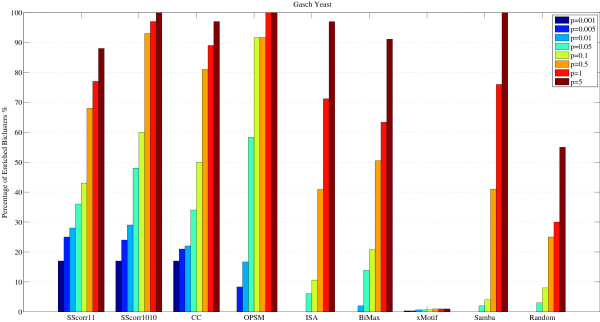
**Comparison of different Biclustering algorithms**. Comparison of different Biclustering methods from Gasch Yeast data set: percentage of enriched biclusters by GO Biological Process category for each method at different significance levels. (p-values from *p *= 0.001% to *p *= 5%).

Figure 7 represents the percentage of enrichment biclusters for each method in which one or several GO terms are overrepresented for different levels of significance (0.001, 0.005, 0.01, 0.05, 0.1, 0.5, 1 and 5). In this Figure, SScorr11 means the proposed Scatter Search with penalization parameters *M*_1 _= 1 and *M*_2 _= 1. Analogously, SScorr1010 is the Scatter Search with *M*_1 _= 10 and *M*_2 _= 10. With p-value *p *= 0.01, the proportion of biclusters significantly enriched by any GO Biological Process category for SScorr11 and SScorr1010 is over 30%, for CC is over 21%, for OPSM over 17%, for BiMax 2% and 0% for the rest. It can be observed that SScorr1010 improves the results of the rest of the methods for small levels of significance except to the CC when *p *= 0.001 (for instance, see the most restrictive level of significance *p *= 0.001 for the p-value). However, both Scatter Search algorithms obtained a percentage of significant biclusters greater than CC for *p *= 0.005 and *p *= 0.01 and the CC presents a percentage of significant biclusters greater than SScorr11 when p-value ranges from *p *= 0.05 to *p *= 5. This is due to the volume of the biclusters since it is easier to find functional enrichment from large groups of genes than from small groups. Table 2 presents information about the size of biclusters obtained by the different methods. Note that biclusters obtained by the CC algorithm have more genes that biclusters for obtained by the algorithms based on Scatter Search. SScorr1010 finds biclusters with more genes than SScorr11 and therefore it improves the results of CC for all levels of significance from *p *= 0.005 to *p *= 5. The rest of methods find a less percentage of biclusters with the p-values specified than the proposed method although OPSM presents good results for high levels of significance (*p >*0.05).

**Table 2:** Comparison of biclusters of different methods

	num. of biclusters	number of genes	number of conditions	volume
**SScorr11**	100	16.4(6.9)	14.8(3.1)	237.6

**SScorr1010**	100	46.7(8.2)	27.1(5.5)	1269.4

**CC**	100	82.0(130.1)	19.8(16.3)	2557.31

**OPSM**	12	95.6(119.6)	12.5(3.6)	849.8

**ISA**	66	76.3(43.9)	8.7(1.4)	645.7

**BiMax**	101	24.0(2.8)	3(0)	72.1

**xMotifs**	306	1.2(0.4)	42.3(11.4)	46.7

**Samba**	100	911.5(132.1)	25.1(8.2)	22344.7

**Random**	100	12.8(2.4)	25.0(2.1)	318.3

Number of genes, average values and standard deviation of number of genes and number of conditions and volume for each method.

**Figure 8:**
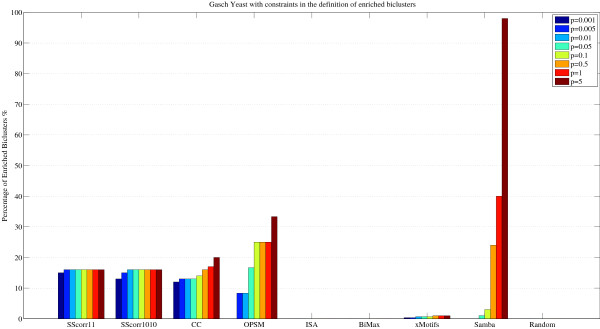
**Comparison of different Biclustering algorithms with hard definition of enrichment bicluster**. Comparison of different Biclustering methods from GaschYeast data set with a restrictive definition of enriched biclusters: percentage of enriched biclusters by GO Biological Process category for each method at different significance levels (from *p *= 0.001% to *p *= 5%) by setting the allowed minimum number of genes per each GO category to 10 and the study fraction greater than 50%.

**Figure 9:**
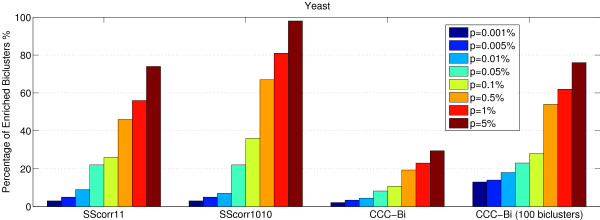
**Comparison of the propsed algorithm with CCC-Bi algorithm**. Comparison between the proposed algorithm based on Scatter Search and CCC-Biclustering [30] for Yeast data set: percentage of enriched biclusters by GO Biological Process category for each method at different significance levels. (p-values from *p *= 0.001% to *p *= 5%).

The comparison is not an easy task because the number of biclusters, their size or what kind of patterns are found are very different for each method. Table 2 presents the number of biclusters for each method, the average and standard deviation for the number of genes and the number of conditions (standard deviation in brackets) and the volume. The standard deviation shows how the size of different biclusters obtained by each method varies. For example CC, OPSM and SAMBA find biclusters with a high number of genes and biclusters with only a reduced group of genes due to the high standard deviation of the number of genes. In order to establish a more restrictive criterion, Figure 7 can be reformulated with the concept of bicluster overrepresented in a GO term as follows. The percentage of enriched biclusters is reported after filtering the biclusters which have less than ten genes in each GO category or have a study fraction less than 50%, that is, there is not more than half of the genes in the bicluster that share the same function in the category [42]. Figure 8 presents the comparison for all methods with this new definition of enriched bicluster. Note that SScorr11 and SScorr1010 obtain the best biclusters for small values of p-value (from *p *= 0.001 to *p *= 0.01) but not for high values from *p *= 0.05 to *p *= 5 where OPSM and Samba present the best results. It can be noted that there is not any significant bicluster for ISA and BiMax algorithms. Figure 9 presents a comparison between algorithms based on Scatter Search (SScorr11 and SScorr1010 with values *M*_1 _= *M*_2 _= 1 and *M*_1 _= *M*_1 _= 10 respectively) and CCC-Biclustering [30] for Yeast data set. Biclusters for the CCC-Biclustering have been obtained using the tool BiGGEsTS [43] and results for all biclusters (CCC-Bi) and for only 100 selected biclusters have been reported. It can be observed that SScorr11 and SScorr1010 obtain a percentage of enriched biclusters greater than that of the CCC-Bi. This fact was expected as SScorr11 and SScorr1010 obtain 100 biclusters and the CCC-Bi obtains 14412 due to this exhaustive method discovers all maximal biclusters in time series gene expression data. Note that if only 100 biclusters of CCC-Bi are considered (the 100 first biclusters that the algorithm reports) the CCC-Bi obtains better results than SScorr11 and SScorr1010 for levels of significance from 0.001 to 0.01 and similar results for p ≥ 0.05.

### 3.4 Biological study

AGO tool [42] has been used to study if a bicluster is composed by a group of genes overrepresented in some GO Biological Processes. The biological study has been focused on the biclusters shown in Figure 3, Figure 4 and Figure 5 whose information is reported in Table 1.

The analysis of *biYeastN15 *identifies the GO process (GO:0006412) with p-value 5.81*e − *006. This process is known as *translation *and it is related with the process in which amino acids are built in the ribosome using mRNA. In this bicluster, 5 genes of the 7 genes are related with this process.

Other example is the bicluster *biLymphomaN1 *which identifies the GO term (GO:0016887) known as *ATPase activity *with p-value 0.0069. This process is related with Catalysis of the reaction of *ATP *molecules.

The study for biclusters from GaschYeast data set discovers the process (GO:0006412) called *translation *for most of the 8 biclusters reported. However, other interesting results are obtained as for example in the bicluster *bi1GaschYeastN11 *where the GO process (GO:0042254) called *ribosome biogenesis and assembly*, with p-value 1.38*e *− 007, which is related with the formation of ribosomas and the transport to the sites of protein synthesis has been found.

## 4 Conclusions

In this paper, a Scatter Search for finding biclusters from gene expression data has been presented. The proposed Scatter Search has used as merit function to evaluate biclusters a measure based on correlations among genes, with the aim of obtaining biclusters with shifting and scaling patterns. Moreover an improvement method, which consist in removing negatively-correlated genes from biclusters, has been incorporated to intensify the search.

The proposed algorithm has been tested with three real data sets: a data set related to yeast cell cycle (Yeast), a data set related to a collection of different stress conditions over Yeast (GaschYeast) and another one related to human B-cells lymphoma (Lymphoma). A group of biclusters composed by genes with shifting and scaling patterns has been discovered some of which can not be detected using the MSR as it was proved in [29]. A comparison using Gene Ontology with other six methods has been presented and a good performance of the proposed algorithm has been observed.

Future works will focus on some improvements for the proposed algorithm with regard to the overlapping among genes and to the fitness function.

## Competing interests

The authors declare that they have no competing interests.

## Authors' contributions

JAN designed and implemented the algorithm, carried out the experimental studies and drafted the manuscript. AT conceived the idea, participated in the design of the algorithm, in the experiments and in the elaboration of the manuscript. JAR motivated the research problem and leaded the project. All authors read, edited and approved the final manuscript.
